# Georgius Hierarchus Simmonsius—The Man in the Glass House

**Published:** 1909-03

**Authors:** G. Frank Lydston

**Affiliations:** Chicago; Professor of the Surgical Diseases of the Genito-Urinary Organs, Medical Department Illinois State University


THE
### TEXAS MEDICAL JOURNAL.
Established July, 1885.

F. E. DANIEL, M. D., - - - - Editor, Publisher and Proprietor.

PUBLISHED MONTHLY.—SUBSCRIPTION $1.00 A YEAR.

VOL. XXIV. AUSTIN, MARCH, 1909. No. 9.

The publisher is not responsible for the views of contributors.

### Original Articles.

### Georgius Hierarchus Simmonsius—The Man in the Glass House.

BY G. FRANK LYDSTON, M. D., CHICAGO.

Professor of the Surgical Diseases of the Genito-Urinary Organs, Medical Department Illinois State University.

It is with some trepidation that I comment on the whilom Esculapian temple of Simmons-the-Great, a photograph of which is herewith appended. It was in this temple, originally dedicated to the service of three ambitious doctors, way out in the sage brush, that Georgius Hierarchus Simmonsius won the eminent scientific and ethical reputation which afterward secured for him the two most eminent positions within the gift of American Medicine. Here he was discovered, first by the reputable physicians of Lincoln, Nebraska, and later by the trustees of the American Medical Association.

*N. B.—The trustees forgot to compare notes with the physicians aforesaid. This statement is both explanatory and apologetic.*

In the city of Lincoln, Nebraska, were published several great newspapers. Of these, none was greater than the *Nebraska State Journal* and the *Evening News.*

Ambitious were these journals, and eke progressive, and for shekels and maravedis did they advertise and depict in their columns the greatest eleemosynary (?) institution of the age. And, by way of good measure, did they advertise the names and fame of three great and shining lights of medicine, whose combined skill did encompass diseases of the skin and of all the tissues and organs that lie within that somewhat elastic specialization of membranous structure. And of these names none shone with brilliancy more effulgent than that of George H. Simmons, L. M., M. D.—now U. S. A., LL. D., A. M. A., Rex.

A talented man, this Simmons, and a modest. Three diverse specialties did he advertise—which proves his manifold talents. He left the world to guess that he was a finished accoucheur, by presenting his "Licentiate in Midwifery" in hieroglyphics. The mystic characters, "L. M.," thus did prove his modesty. The delicacy with which he combined diseases of the skin, genito-urinary diseases and gynecology was highly commendable.

And the "Institute" of that day was of brick and stone and wood, and, though it "failed" most grievously, did not fall about the ears of Simmons.

But, as time went on, and Simmons grew in importance and degrees, he builded him a new temple on Dearborn Avenue in Chicago, with the magic symbol "A. M. A." above its portals, and, feeling himself secure within it, did throw stones, as 'twere from a mighty catapult, at those whom he suspected of being of his former ilk, little thinking that the new house was built in the verisimilitude of the old, but with roof and walls and doors of glass. And he threw many large and heavy bolts and rocks at the unethical brethren of his former days so recklessly that he struck not only his ethical equals, but likewise his betters. And every time his deadly catapult snapped, he smashed a window or a transom, or eke a door in his own house.

Deluded by his egotism, drunk with power and dazzled by the groveling obsequiousness of his hirelings and political bedfellows, he kept on with his roof and wall and window smashing, until naught was left but frames and sashes, and there was no longer a roof to cover him nor walls to shield him from the elements. And before many moons had passed the wayfarer who chanced to fare by the house of glass beheld a solitary, woe-begone, shivering figure within it, half buried under a ton of hieroglyphs.

And it came to pass that Georgius Hierarchus Simmonsius did sell for the wherewithal to sustain him those hieroglyphs to the ragman. And now the ragman's wife, as she walks in all her glory abroad o' Sundays, doth shine resplendant in a gown of many colors of "changeable" silk, on which blaze many mystic characters, the which the scribes and priests can not decipher, the same being "Lieut., U. S. A., A. M., M. D., L. M., LL. D."

And in the ash heap in the alley, behind the ruined house of what was once glass, reposes a tarnished crown of tinsel, on which dully gleams the mystic symbol "A. M. A., Rex."

APPENDIX.

Lest it seem that I have published the story of the glass house surreptitiously, without forewarning the distinguished occupant thereof, I herewith append a series of letters which will relieve the reader's mind upon that point:

CHICAGO, December 13, 1908.

*Dr. George H. Simmons,* 103 *Dearborn Ave., City.*

DEAR SIR: I am engaged in the preparation of an article on medical politics. Obviously, the editor of the *Journal* will come in for a share of attention. Incidentally, I would like to know whether a statement made to me is true, viz., that for some years you were connected with a so-called "institute" which was advertised in the secular press and in which advertisements your name appeared as a specialist in several departments of medicine.

An early answer will oblige,

Very truly yours,

G. FRANK LYDSTON.

CHICAGO, December 15, 1908.

*Dr. G. Frank Lydston, Chicago, Illinois.*

DEAR DOCTOR: I do not quite understand the object of your letter of the 13th instant, just received. I have been informed that you, Abbott, Lamphear, et al., are intending to put in a new editor, that detectives have been diligently working in Lincoln for material, and that, among other things, advertisements of the old Lincoln Medical Institute have been resurrected. If this is true, then you know more about those advertisements than I do, for it is so long ago that I have forgotten practically all about them.

Of course, I may have been mistaken—may have been misinformed; it is possible that you really want the actual facts connected with this hospital—for that was what this "institute" was, and a very well conducted hospital, too—and have written me for information that you do not have.

As I am an interested party, I am forwarding your letter to a Lincoln physician, who, I think, has more knowledge than I as to what the advertisements were, and have requested him to write to you.

Very truly yours,

GEORGE H. SIMMONS.

It will be noted that I did not in my letter of inquiry asperse the character of the work done in the Lincoln Medical Institute. It would be difficult to secure data upon that point—in evidence of which note the omnibus character of its scope, as shown by its ads and the multiform specialties of its staff—the Medical Trinity magnificent and omniscient. Especially am I inhibited in my criticism by my ignorance of the "water cure' and of "compound oxygen," the *ultima thule* of quackery.*

Had Simmons one per cent of the wisdom and acumen he affects, he would not—suspecting that I already knew the facts—have answered my letter as he did. His cue would have been either to tell me to go to blazes or ignore the letter altogether. But, alas! for his perspicacity, he grasped at the frailest of straws—a friend at a distance. But his letter was characteristic of the man—extra large in type and couched with all the dignity of a schoolboy caught making faces at his teacher.

I answered the gentleman's letter in this wise:

CHICAGO, December 16, 1908.

*Dr. George H. Simmons,* 103 *Dearborn Ave., City.*

DEAR SIR: As you seem to be posing as a mind reader, I will endeavor to set you straight. So far as I am concerned, I am trying to get at the truth regarding the point in question. As to "Abbott, Lamphear, et al.," their welfare or politics do not interest me, save in so far as they may involve a principle, any evidence they may provide as to my own medico-political views will be received and weighed by me as impartially as evidence received from any other source. As to my being a party to alleged "detective work" in Lincoln, Nebraska, whoever gave you information to that effect deliberately lied. As you seem to have been informed of an alleged conspiracy to get you out of your job, I will set you right as to myself. I hold the opinion that you are intellectually and morally unfit for the positions you now hold, and consider your appointment to them an insult to the intelligence of the regular profession. I doubt if any one could dethrone you—you are too clever and wily a politician—but if I

*It is hardly necessary to reminl the profession of the past popularity with the quacks of the "compound oxygen" fake.—G. F. L.

could feel that any word or act of mine had contributed to sucn a fortunate event, I should feel that I had not lived in vain.

Very truly yours,

G. FRANK LYDSTON.

When I received the letter from his former partner, for which Simmons wrote to Lincoln, I was somewhat surprised to note that my questions had been answered affirmatively by Dr. Garten. ("Save us from our friends.") Here, again, note the defense of the character and scope of the work done by the hospital—a point absolutely irrelevant to my inquiry. The writer states that Simmons need not be ashamed of his connection with the work of the hospital in the field of medicine. (Be it remarked that I never have attempted to "shame" Simmons.) My vocabulary and argumentative talent have their limitations. Neither have I criticised in any way the work which the "Institute" did in the field of medicine.

LINCOLN, NEBRASKA, December 17, 1908.

*Dr. G. Frank Lydston, Chicago.*

DEAR DOCTOR: I have a letter from Dr. Geo. H. Simmons accompanied by one over your signature, which he requests me to answer.

Dr. Simmons was connected with the Lincoln Medical Institute, a hospital whose doors were open to all physicians and their patients. At that time it was the only hospital in the State of Nebraska outside of Omaha. If it committed a sin, it was only in the fact that *it did advertise in the secular press.*

More than twenty years have passed since its doors were closed. Simmons was resident physician during one year or maybe a little longer. (At its ending.)

I do not remember that he advertised as a specialist in several departments of medicine, but *he did advertise as one giving special attention to genito-urinary organs. Yet what he did in this line is a matter of record and can be had from the files of the State Journal and the Evening News, two papers published here at that time.*

So far as the Lincoln Medical Institute is concerned, he need not be ashamed of his connection with its work in the field of medicine.

*Along this line I know as much as any one, being connected with it from first to last.*

Very respectfully yours,

Italics mine.—G. F. L. M. H. GARTEN.

And Simmons has forgotten! Ye gods of those dark recesses of the mind wherein memory sits enthroned, forgive this man his evasion of the issue! Members of the A. M. A., believe not that this, our puissant, omniscient master, stands self-confessed of premature and doddering senilescence.

Remember? Aye, clearly dost thou remember the picture and the ads. that appeared in those secular journals in the days when thou wast not preaching ethics nor hadst the power to crush all whose personality or policies did not please thee.

Remember? Aye, well and passing well. Those ads. are seared in everlasting brand upon the very marrow of thy brain, and like all-searching eyes of relentless demons, those ads. of specialties and of titles have disturbed thy dreams—have turned pleasant visions of more and yet more place and power into horrid nightmares. They have caused the shadowy spectral shapes of shekels and maravedis, which have been the *summum magnum* of thy sordid life's ambition, to elude thine eager fingers even as the greased pig eludes the yokels at that scene of bucolic festivity yclept ye county fair. Ever in the still watches of the night the genii who dwell within that well of obscurity in which thou fain wouldst bury thine unethical past—that past which thou wouldst not that humble lesser lights should know—doth menace thee with dire threats of publicity of thy non-ethics of aforetime. And when the giant harlequin who beareth unpleasant dream pictures leaps upon thy nocturnal stage, bearing sheaves of embarrassing recollections of weirdly multiple specialties and of "compound oxygen," thou find'st cold comfort in the ethical whitewash with which self-interest hath daubed thee in these thy later and fatter years. And comfort colder still wilt thou derive from those shafts of malice which thou hast sped at ethical sinner and ethical innocent alike, in these thy days of hypocritic reconstruction that ne'er can reconstruct.

Tell me, O man of evasiveness and disingenuous guile, hast thou not been for years and years afeared that thou wouldst one day tempt the fates too far and flaunt just once too often thine egotism and abuse of power in the face of the goddess Fortune? Yea, verily, for thou are wise, according to thy lights and after thy selfish fashion, and the dread spectre of thy flagrant irregularity of other days thou canst not down. Like Banquo's ghost, the remembrance of the time when thou didst brag too much and advertise more, hath haunted thee, and smitten thee till thy very liver hath wobbled in its function—although, forsooth, be it here and now confessed that thou ne'er hast been in danger of famine of the gall.

*And this is the man who is the head and front of American medical journalism; who dictates our policies, controls medico-political appointments, supervises our organization, censors our articles, handles the business of our great journal, tells us what there is of value in our armamentarium therapeuticum, tells us what shall be advertised and what shall not, supervises the ethics and morals of our drug manufacturers, tells our independent medical journals what they shall advertise and what they shall not, receives invitations to lecture before our medical societies on the proper methods of teaching therapeutics—in short, who is the arbiter of all things literary, ethical, political, therapeutic and moral in American medicine!*

